# Current News

**Published:** 1904-10

**Authors:** 


### Current News.

### INTERNATIONAL DENTAL CONGRESS—REPORT OF COMMITTEE ON PRIZE ESSAYS.

To the International Dental Congress:

The committee to whom was given the consideration of papers competing for the prize offered by the Committee of Organization begs leave to make its report to the Congress assembled at St. Louis.

Your committee decided upon the following rules for its government in considering papers presented:

1. The subject-matter must be new and original.
2. The paper must not be overloaded with quotations.
3. It must, in every respect, be in accord with the character and dignity of the Congress.

Ten papers have been presented and carefully considered. Several of these are so nearly up to the standard adopted that your committee has found some difficulty in reaching a united conclusion. It has, therefore, been regarded as proper and just, in deciding upon the paper worthy of the prize, to mention several equally worthy of "Honorable Mention."

The paper entitled "A Study of Certain Questions relating to the Pathology of the Teeth," prepared by Professor W. D. Miller, of Berlin, is, in the opinion of your committee, entitled to the highest honor that this Congress can bestow.

The prize, therefore, is conferred upon his work.

The paper entitled "The Pathology of Lime Salts in Nutrition," by Dr. Med. C. Röse, of Dresden, Germany, is regarded by your committee as one of the most thorough and valuable papers ever prepared upon this subject. It presents years of patient work, but unfortunately does not and, as yet, cannot go beyond clinical observation.

Your committee, therefore, report for "Honorable Mention" in the order of merit, the following:

1. "The Pathology of Lime Salts in Nutrition," Dr. Med. C. Röse, Dresden, Germany.

2. "Constitutional Causes of Tooth-Decay, Erosion, Abrasion, and Discoloration," Dr. Eugene S. Talbot, Chicago, Ill.

3. "Anatomic Changes in the Head, Face, Jaws, and Teeth in the Evolution of Man," Dr. Eugene S. Talbot, Chicago, Ill.

4. "The Development of the Teeth of the *Sus Domesticus,*" Zahnarzt Max Hirsch, Halle, Germany.

The balance of the papers, valuable for section work, have been transferred to the proper custodians.

Two papers were sent in for examination, but both too late to compete for the prize. One by Dr. Michaels, of Paris, not in the hands of the committee, and title unknown, and one entitled: (1) "Investigations concerning the Corrosibility of Aluminum," and (2) "Applicability of Aluminum to Dentistry," a very full paper upon this subject, by Hof-Zahnarzt W. Pfaff, Dresden, Germany.

(Signed) WILBUR F. LITCH,
L. M. COWARDIN,
JAMES TRUMAN, *Chairman.*

ST. LOUIS, MO., August 29, 1904.

---

### NATIONAL ASSOCIATION OF DENTAL EXAMINERS—REPORT OF COMMITTEE ON COLLEGES.

IT will be remembered that the relations between the National Association of Dental Faculties and our Association which had obtained for some time during the past were left intact at the close of the meeting of this Association at Asheville last year. It will be unnecessary to more than briefly refer to the things that have transpired in the National Association of Dental Faculties since that meeting. Suffice it to say that that body has been wrestling with itself as to whether it would, or even could, keep faith with the public and with this Association in the carrying out of the requirements of course for graduation which it had definitely set up in

1901 and put into actual operation beginning with the school year 1903–04.

It transpires that at least a majority of that Association, by official action, has seen fit, for reasons they consider paramount, to seriously modify their course requirements for graduation in a way and to a degree that your committee, as well as the profession at large, cannot interpret in any other way than as a most deplorable retrogression. It would appear, from what has thus occurred, that the National Association of Dental Faculties has clearly demonstrated that, no matter in how good faith it entered upon the establishment of the new standard in 1903–04, it was utterly powerless of its own unaided strength to permanently establish and maintain it.

After a careful earnest canvass of the whole situation by correspondence and interview with many of the leading dental educators of this country, as well as with many leading and influential practitioners of experience and observation, and also backed by some of the best legal advice procurable, as to what was the duty of this deliberative body, composed as it is of the various State boards, endowed under their various laws with the judicial and discriminative power and duty of establishing and maintaining reasonable dental educational standards and requirements, your committee is fully convinced that the time is ripe for this Association to declare in no uncertain tones what we hold to be most essential and necessary qualifications of a prospective dental college student in order to enable him to assimilate and appropriate the great fund of scientific knowledge offered him in any of our properly conducted dental schools.

Your committee would therefore recommend that this Association establish at once, to go into operation not later than the opening of the school year of 1905–06, the educational requirement for admission to the dental college course of graduation from an accredited high school or its full equivalent, all examination of credentials and equivalents to be placed in the hands of an acceptable appointee of the State Superintendent of Public Instruction where not otherwise provided for by law.

In view of the present disturbed and unsettled conditions existing in dental educational circles, and with a belief in the wisdom of avoiding all unnecessary disturbance of standards at this time, your committee would further recommend that no change be made at this time in the present requirements of this Association of not less than twenty-eight calander months of college attendance for graduation.

Charles C. Chittenden, *Chairman,*
H. J. Burkhart,
J. A. Hall,
*Committee on Colleges of the N. A. D. E.*

Unanimously adopted by the National Association of Dental Examiners at St. Louis, Mo., August 27, 1904.

---

### NEW JERSEY STATE BOARD OF REGISTRATION AND EXAMINATION.

The New Jersey State Board of Registration and Examination in dentistry will hold their semiannual meeting in the Theoretical branches, in the Assembly chamber at the State-House, Trenton, N. J., on the 18th, 19th, and 20th of October, 1904. Practical prosthetic work to be done in the office of Dr. A. Irwin, 425 Cooper Street, Camden, N. J., and practical operative work to be done in the office of Dr. C. S. Stockton, 7 Central Avenue, Newark, N. J. Work to be done on dates assigned by the Examiner in those branches.

All applications must be in the hands of the secretary by the 15th of October.

Charles A. Meeker, D.D.S.
29 Fulton Street, Newark, N. J.

---

### NORTHERN INDIANA DENTAL SOCIETY.

The sixteenth annual meeting of the Northern Indiana Dental Society will be held October 18 and 19, at Huntington, Ind.

It is expected that we will have the largest attendance of any meeting ever held in this section of the country, and you cannot afford to miss hearing such essayists as Drs. G. V. Black, Hart J. Goslee, F. E. Roach, George E. Hunt, William T. Reeves, E. X.

Jones, J. Q. Byram, G. E. Johnson, F. R. Henshaw, F. M. Bozer, Lavinia B. McCollum, C. G. Keehn, and many others that have consented to appear on the programme, besides a very attractive list of clinics demonstrating all of the newest and most valuable things in practice.

All the leading manufacturers have signified their intention of making an exhibit of their products.

Every up-to-date dentist will be present. Are YOU coming?

Special social features for Tuesday evening.

Remember the date.

OTTO U. KING,
*Secretary.*

---

### ARKANSAS STATE BOARD OF DENTAL EXAMINERS.

THE next meeting of the Arkansas State Board of Dental Examiners will be held December 2 and 3, 1904, in Little Rock, Ark., for the examination of all applicants. Those having applied for examination will report to the secretary Friday morning, December 2, 1904, with rubber dam, gold, plastic filling-material, and instruments, to demonstrate their skill in operative dentistry. Any one who wishes may bring a patient; as far as possible patients will be furnished. The board reserves the right to select the cavity to be filled.

The examination will cover all branches of the dental profession.

No temporary certificate will be issued to any one. Examination fee, $5.00. For further information write the secretary.

DR. A. T. MCMILLIN,
*Secretary.*

